# Optimized Intelligent Classifier for Early Breast Cancer Detection Using Ultra-Wide Band Transceiver

**DOI:** 10.3390/diagnostics12112870

**Published:** 2022-11-19

**Authors:** Ahmad Ashraf Abdul Halim, Allan Melvin Andrew, Wan Azani Mustafa, Mohd Najib Mohd Yasin, Muzammil Jusoh, Vijayasarveswari Veeraperumal, Mohd Amiruddin Abd Rahman, Norshuhani Zamin, Mervin Retnadhas Mary, Sabira Khatun

**Affiliations:** 1 Advanced Communication Engineering (ACE), Centre of Excellence, Universiti Malaysia Perlis (UniMAP), No. 15 & 17, Jalan Tiga, Pengkalan Jaya Business Centre, Kangar 01000, Perlis, Malaysia; 2 Faculty of Electronic Engineering & Technology, Universiti Malaysia Perlis (UniMAP), Pauh Putra Campus, Arau 02600, Perlis, Malaysia; 3 Advanced Computing (AdvCOMP), Centre of Excellence, Universiti Malaysia Perlis (UniMAP), Pauh Putra Campus, Arau 02600, Perlis, Malaysia; 4 Faculty of Electrical Engineering & Technology, Universiti Malaysia Perlis (UniMAP), Pauh Putra Campus, Arau 02600, Perlis, Malaysia; 5 Department of Physics, Universiti Putra Malaysia, Serdang 43400, Selangor, Malaysia; 6 College of Computing, Saudi Electronic University (SEU), Riyadh 13316, Saudi Arabia; 7 Faculty of Electrical and Electronics Engineering Technology, Universiti Malaysia Pahang (UMP), Pekan 26600, Pahang, Malaysia

**Keywords:** feature selection, prediction, feature engineering, multi-stage, machine learning, supervised learning, breast cancer

## Abstract

Breast cancer is the most common cancer diagnosed in women and the leading cause of cancer-related deaths among women worldwide. The death rate is high because of the lack of early signs. Due to the absence of a cure, immediate treatment is necessary to remove the cancerous cells and prolong life. For early breast cancer detection, it is crucial to propose a robust intelligent classifier with statistical feature analysis that considers parameter existence, size, and location. This paper proposes a novel Multi-Stage Feature Selection with Binary Particle Swarm Optimization (MSFS–BPSO) using Ultra-Wideband (UWB). A collection of 39,000 data samples from non-tumor and with tumor sizes ranging from 2 to 7 mm was created using realistic tissue-like dielectric materials. Subsequently, the tumor models were inserted into the heterogeneous breast phantom. The breast phantom with tumors was imaged and represented in both time and frequency domains using the UWB signal. Consequently, the dataset was fed into the MSFS–BPSO framework and started with feature normalization before it was reduced using feature dimension reduction. Then, the feature selection (based on time/frequency domain) using seven different classifiers selected the frequency domain compared to the time domain and continued to perform feature extraction. Feature selection using Analysis of Variance (ANOVA) is able to distinguish between class-correlated data. Finally, the optimum feature subset was selected using a Probabilistic Neural Network (PNN) classifier with the Binary Particle Swarm Optimization (BPSO) method. The research findings found that the MSFS–BPSO method has increased classification accuracy up to 96.3% and given good dependability even when employing an enormous data sample.

## 1. Introduction

Breast cancer is the most common cancer worldwide and the leading cancer compared to other types of cancer for women [1]. It is the fifth most-frequent cancer that causes death in women, especially in developing countries, where screening systems are limited and sometimes nearly non-existent [2,3]. Previous studies have stated that early breast cancer detection or screening and accurate diagnosis and treatment could improve long-term breast cancer survival rates while lowering treatment costs [4]. Note that cancer is a condition in which the body replicates cells and cell responses are out of balance, resulting in abnormal cell growth or a tumor. Note that the tumor is either benign (noncancerous) or cancerous (malignant). Benign tumors do not spread to other body parts or invade neighboring tissues (metastasize) [5]. On the other hand, a malignant tumor is made up of cancer cells that can penetrate and damage surrounding tissues and affect different body sections. Other than that, chronic problems can occur if cancer cells move to other organs. Therefore, it is pretty apparent that early detection of the cancer cell’s presence is crucial to cure and prevent the cell from spreading to the other part.

**Figure 1:**
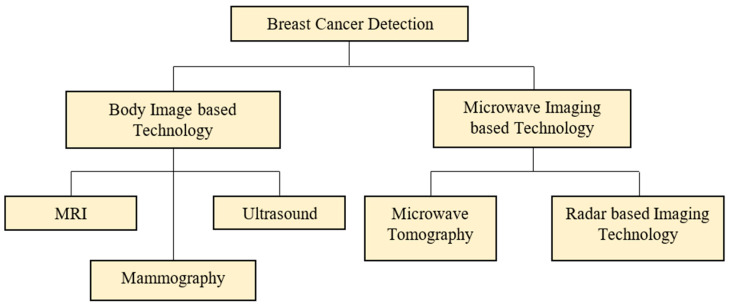
Breast cancer screening technology [18].

Numerous existing screenings and developing technology are used to diagnose breast cancer early in its stages [6]. Current breast-cancer-screening technologies are divided into two groups, as shown in Figure 1, body imaging-based technology and microwave imaging-based technology. Magnetic Resonance Imaging (MRI), mammography, and ultrasound are examples of body image-based technology that obtain the breast structure images to be reviewed and evaluated by the radiologist [7,8]. Most clinics and hospitals have these tools on hand. On the other side, microwave imaging-based technology has the alternative to replace costly and invasive screening procedures [9,10,11,12]. Furthermore, this technology is safe, durable, free of ionizing radiation exposure, and causes users less physical harm [13,14]. Microwave tomography and radar-based imaging are two approaches used in microwave imaging technologies [15,16,17]. The Ultra-Wideband (UWB) signals were employed in both ways to categorize breast cancer based on its dielectric properties. The main contributions of this research work are to modify the hybrid statistical feature generator model for optimized feature selection to improve classification accuracy and propose a complete design framework for early breast cancer detection.

The rest of this paper is structured as follows: the related works, materials and method are described in Section 2 and Section 3, respectively, which propose a multi-feature selection technique in detail. The results and discussions are presented in Section 4, while Section 5 concludes the study.

## 2. Related Works

Many other researchers have conducted studies on breast cancer detection using the Ultra-Wideband (UWB) [19]. This includes Khondker Jahid Reza et al. [20], who proposed an early breast cancer detection technique by developing a system integrating a small-size UWB biomedical antenna and feature extraction technique for the Artificial Neural Network (ANN) [21], in which the system can detect tumor existence and measure the size. Forward scattering signals comprise four characteristic features for pattern recognition and tumor signature investigation, including maximum, minimum, average, and standard deviation.

On the other hand, Nouralhuda et al. [22] proposed a computational method for the detection of breast tumors using UWB microwave technology. The proposed technique uses ANN feedforward backpropagation for detecting and recognizing tumors based on the dielectric properties of human mammary tissues. The research used a sample of a fixed tumor-sized diameter of 2.5 mm and was placed in various locations. Note that the database consists of only 118 datasets, with a single feature extraction using Fast Fourier Transform (FFT) to classify the tumor in the breast phantom. However, they could only demonstrate one tumor size during the procedure. Therefore, various tumor sizes are recommended for data analysis to mimic the actual tumor in the breast phantom, thus making more accurate predictions.

According to work published in recent years, R.C. Conceicao et al. [23] presented a classification of breast tumor models of varying sizes and shapes using signals collected with a monostatic UWB radar microwave imaging prototyped with machine learning algorithms. The classification was evaluated with Principal Component Analysis (PCA) as a feature extraction method and tuned Naïve Bayes (NB), Decision Tree (DT), and k-Nearest Neighbor (kNN) as the classifier.

Bifta et al. [24] proposed an Artificial Neural Network (ANN) technique with single-stage feature extractions using small data samples. Hence, more data samples needed to be collected and tested through the proposed statistical feature generator method to prove it can perform well for the larger-size dataset. Their paper investigated early breast cancer detection based on UWB hardware and used a Feedforward Backpropagation Neural Network (FFBPNN) in three dimensions with the “feedforward net” function. This paper only discussed a single feature extraction method to minimize the data size feature from 1632 data points to only 4 data features before it moved to ANN for classification. A vast number of valuable data will be lost during the procedure, and it is suggested to have an MSFS to ensure that only essential data will be processed during the training procedure.

Vijayasarveswari et al. proposed a Multi-Stage Feature Selection (MSFS) method that extracts significant features statistically for breast cancer size detection using data normalization techniques with 6750 data samples [25]. Note that the proposed algorithm has four parts—comprising data normalization methods, feature extraction, dimensional reduction, and feature fusion. The output is fused to generate different datasets, namely, 8-HybridFeature, 9-HybridFeature, and 10-HybridFeature datasets. The classification performance of the datasets is tested using the Support Vector Machine (SVM), Probabilistic Neural Network (PNN), and NB classifiers for breast cancer size classification. The research findings discovered that the 8-HybridFeature dataset performs better than the other two datasets, although it has specific statistical feature analysis in terms of the complete framework. The summary of the previous study on breast cancer detection using UWB is shown in Table 1.

The capabilities of a machine learning model depend on the characteristics utilized during training. The selection of the characteristics is based on the diverse approaches to feature selection offered by different researchers. According to the prior study, the standard feature selection approach adopted by researchers is essentially a single-stage method. Typically, researchers collect features by extraction, selection, or normalization. This strategy, however, adds to an increase in the misclassification rate due to inadequate data processing. In addition, the exploration and exploitation of the data are insufficient during the feature selection, since the features are decreased based on the starting condition, causing the selected features to be redundant or some beneficial characteristics to be lost. Exploration involves the discovery of characteristics through a multi-stage process, whereas exploitation entails the addition of relevant information to the prior optimal solution.

This work is motivated by discovering and developing an MSFS–BPSO strategy that provides an efficient machine learning model. Traditionally, a collection of robust features is chosen following data analysis to prevent the creation of an overfitted machine learning model. Based on past research, some researchers pick the subset of features based on the machine learning score, while others select them during the creation of the machine learning model. However, the selected features may not be suitable to various machine learning model types since feature selection relies highly on machine learning. If the same feature is utilized for several forms of machine learning, it increases the likelihood of developing models with a high misclassification rate. Determining characteristics by employing the inherent attributes of the data with tremendous significance and the slightest similarity is thus an additional objective of this study. This can be accomplished by rating the traits to determine their relative value. This allows the model’s complexity to be lowered and the optimization problem to be addressed.

One of the main goals of breast cancer detection research is to create a thorough framework for the identification of cancer. Developing a comprehensive framework for breast cancer detection is one of the primary concerns in breast cancer detection research. Only a few researchers can establish a framework from data sample collection to visualization to identify breast cancer in their studies. Only a few researchers can set up an entire framework, from data sample collecting to visualization, to identify breast cancer in their studies. Shirazi (2017) [29], Huang (2017) [30], and R. Chtihrakkannan (2019) [31], for instance, developed a framework to determine the presence or absence of breast cancer.

In contrast, Santorelli (2014) [32], Liu (2021) [27] and Lu (2022) [26] developed a breast cancer detection and localization framework. Reza (2015) [33] and Vijayasarveswari (2020) [25] merely provided a framework to estimate the breast cancer’s size, whereas Chaurasia (2018) [34], M. Islam (2020) [35], and B. Kharthikeyan (2020) [36] only created a framework to research the different types of cancer (benign and malignant). However, for the most part, researchers are looking at how to create a system that can recognize every breast cancer symptom. As a result, it’s essential to create a comprehensive framework for breast cancer screening that incorporates many early detection criteria.

Although many single-stage feature techniques and classifiers have been proposed, the optimized MSFS for breast cancer detection using UWB has yet to be discovered. This paper investigated an MSFS method optimized with BPSO and singular value decomposition for data reduction that provides the highest detection accuracy, minimizes misclassification, and promotes high breast cancer detection reliability. This research is essential for determining the better version of statistical features and classification algorithms that have the potential to be used in breast cancer detection, including existence, size, and location.

## 3. Materials and Methods

This section presents the materials and experimental methodology used in this project. The process started with data collection, which comprised material used for breast phantom and tumor development. Next, the hybridization of the Multi-Stage Feature Selection with Binary Particle Swarm Optimization (MSFS–BPSO) framework is divided into six stages: (a) feature normalization is to convert the feature into the same scale, (b) feature dimension reduction, which is used to transform the data from a high dimensional space into a low dimensional space without losing important properties of the original data, (c) feature selection (based on time/frequency domain) is used to set the best group result between the time domain and frequency domain, (d) feature extraction is used to shrink the number of features in a dataset by creating new features from the existing one, (e) feature selection (optimization) identifies an optimized feature set, and (f) finally, feature fusion combines different features from different layers. Note that only one type of domain is selected to continue the process.

### 3.1. Breast Phantom and Tumor Development

Various breast phantoms have been proposed to explore the researcher’s capability to detect breast cancer [37]. According to literature studies, most researchers employ low-cost and non-chemical substances such as petroleum jelly, a blend of wheat flour, water, and soy oil, to create heterogeneous breast phantoms [38]. It is important to ensure that the breast phantoms possess permittivity and conductivity values comparable to actual breast tissue, as shown in Table 2.

**Figure 2:**
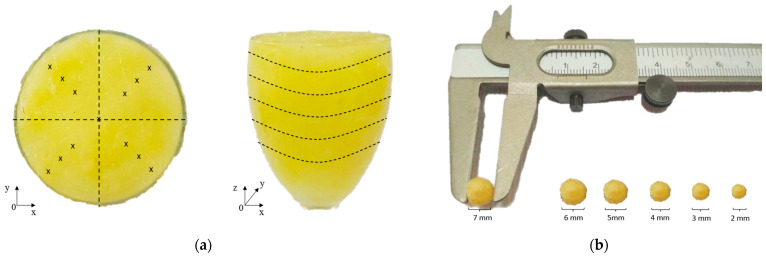
(**a**) The breast phantom; (**b**) The tumor. Figure shows the developed breast phantom and tumor for the experiments.

The breast phantom comprises a 75 mm wide, 60 mm high, and 1.9 mm thick hemispherical wine glass that serves as the skin. Consequently, it is placed into the phantom for each experimentation trial in a new spot. Pure petroleum jelly serves as the breast fatty tissue used in this research. Meanwhile, the tumor is made from a mixture of 10 g wheat flour and 5.5 g water (10:5.5), as shown in Figure 2a,b, respectively.

### 3.2. Experimental Setup

The proposed system architecture consists of hardware and software modules. The hardware includes two antennae (transmitter and receiver), a breast phantom, a tumor, and an Ultra-Wideband (UWB) transceiver with a Personal Computer (PC) interface. Here, the software comprises a data processor, classifier, and Graphical User Interface (GUI).

**Figure 3:**
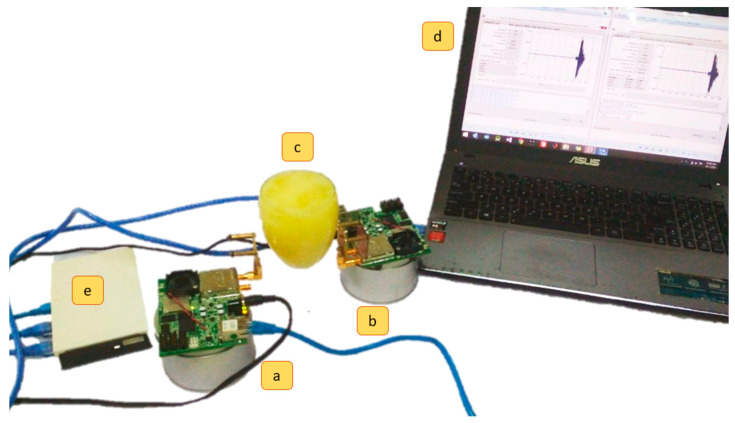
Experimental setup for breast cancer detection: (**a**) Transmitter; (**b**) Receiver; (**c**) Breast Phantom; (**d**) computer; (**e**) router.

As illustrated in Figure 3, the heterogenous breast phantom is placed between the transmitter and receiver. The Ethernet cable connects the router to the UWB transceiver (P400 RCM). Then, UWB pulses were created in the transceiver and transferred through the transmitting signal to the receiver. Correspondingly, the receiver antenna then captured the signals at the center frequency of 4.3 GHz, passing through the router before all the data were analyzed using Matlab software [39,40]. The experimental design adopted in this study is comparable to the technique used in the studies detailed in [24,25].

The steps for collecting data are as follows:

1. The 2 mm tumor is implanted in a heterogenous breast phantom;
2. The single transmitting antenna (Tx) transmits UWB signals, and the opposite single receiving antenna (Rx) captures forward scattered UWB signals. Fifty repetitions are taken for each cycle;
3. The tumor is placed in 65 different locations within the breast phantom. Each tumor (of the same size) is placed at different locations using the combination location of x coordinates (1 cm, 2 cm, 3.35 cm, 4 cm, 5 cm, 6 cm), y coordinates (1 cm, 2 cm, 3.35 cm, 4 cm, 5 cm, 6 cm), and z coordinates (4 cm, 5 cm, 6 cm, 7 cm, 8 cm);
4. Steps 1 to 3 are repeated until all the locations in the breast phantom are covered. The tumor size is then changed to other sizes (3 mm, 4 mm, 5 mm, 6 mm, and 7 mm);
5. For no-tumor data, the breast phantom will rotate 360 degrees (with 60 different angles). Three hundred twenty-five repetitions are taken for each cycle.

A sample of forwarding scattered time domain signals were transmitted and received. A total of 39,000 UWB signals were collected, with each signal sample having 1632 data points. Typically, the signal is in the time domain. In the time domain, the signal characteristics are simpler to see. However, assessing the signal characterization in the frequency domain is equally crucial since it enables the observation of the signal’s properties that cannot be seen in the time domain. As a result, the time domain signals collected from the UWB transceivers were converted to frequency domain signals using the widely utilized Fast Fourier Transform (FFT). The signal’s maximum peak occurs roughly around 4.3 GHz, which is also the operating frequency of the UWB antenna.

### 3.3. Multi-Stage Feature Selection

**Figure 4:**
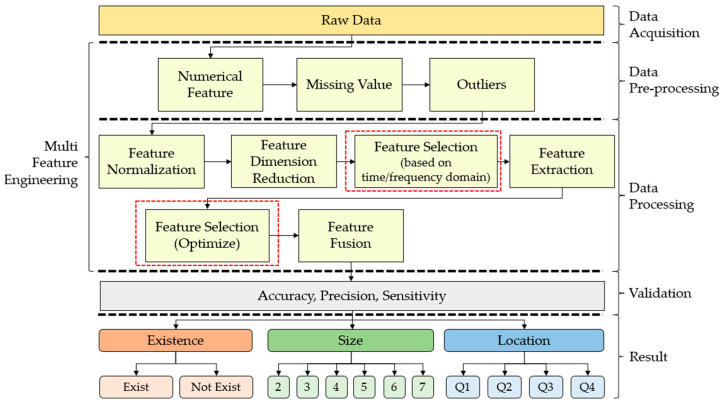
MSFS–BPSO flowchart of overall experimental process.

The process of creating new input features for machine learning is known as feature engineering, which extracts the features from raw data. The presence of the proper feature characterizes successful machine learning algorithms. Other than that, these characteristics are then converted into formats suitable for the machine learning procedure. Data-specific expertise is essential to the process. The overall flow chart is shown in Figure 4. It is summarized into five stages: (a) data acquisition, (b) data pre-processing, (c) data processing, (d) validation, and finally, (e) results.

Multi-Stage Feature Selection (MSFS) consists of data pre-processing (handling numerical features, missing values, and outliers) and data processing (feature normalization, feature dimension reduction, feature selection classifiers, feature extraction, feature selection, and feature fusion). The importance of MSFS–BPSO is to reduce complexity and increase accuracy. Apart from that, this system was trained and tested using a total of 39,000 data samples. A total of 60% of data samples were used for training, 20% for testing and the remaining 20% for validation. The received signals were processed to provide 1632 discrete data points for each sample.

#### 3.3.1. Feature Normalization

Feature normalization is a technique used for standardizing the range of features without reducing the data dimension. The normalization of pre-processed data is essential because it is essential to select the best characteristics without excluding useful information. Consequently, this study analyzed raw data samples using five distinct data normalization techniques. Based on a comprehensive review of previous research, five commonly used feature normalization methods were selected: the Binary Normalization (BN), Decimal Scaling (DS), Z-score (ZS), Linear Scaling (LS), and Min–Max (MM) methods, as mentioned in Equations (1)–(5).

The BN normalization method rescales the data from one range to a new range, the [0, 1] range, using Equation (1), where v is the current value, *v_max_* and *v_min_* are maximum and minimum value of normalized data.

(1) $$\mathrm{Binary}\;\mathrm{normalization},\;v\prime_{BN}=\frac{0.8\left(v-v_{min}\right)}{v_{max}-v_{min}}+0.1.$$

The DS method normalizes the data by moving the decimal points. The number of decimal points depends on the maximum absolute value of the data sample, *D*. It is computed using Equation (1), where v is the instantaneous value of feature *D*, and *j* is the smallest integer that can obtain a maximum $v^{\prime }$ with a value less than 1.

(2) $$\mathrm{Decimal}\;\mathrm{scaling},\;v\prime_{DS}=\frac{v}{10^{j}}\;.$$

LS is the case of the MM normalization method. It normalizes the data to a [0, 1] range based on Equation (3), where v is the instantaneous value of feature *D* and *max_D_*, and *min_D_* are the maximum and minimum values of D, respectively.

(3) $$\mathrm{Linear}\;\mathrm{scaling},\;v\prime_{LS}=\frac{v-min_{D}}{max_{D}-min_{D}}.$$

The MM normalization method rescales the data from one range to a new range, the [−1, 1] range, using Equation (4), where v is the instantaneous value of feature *D*, *max_D_* and *min_D_* are the maximum and minimum values of *D*, respectively, *new_max_D_* is one and *new_min_D_* is −1.

(4) $$\mathrm{Min}-\max,\;v\prime_{MM}=\frac{v-min_{D}}{max_{D}-min_{D}}\left(new\_max_{D}-new\_min_{D}\right)+new\_min_{D}.$$

The data are normalized by converting the value to a common scale with zero mean and unity standard deviation, as shown in Equation (5). Here, v is the instantaneous value of feature *D*, while ${µ}_{D}$ and $\sigma_{D}$ are the mean and standard deviation of feature *D*, respectively.

(5) $$Z-\mathrm{score},\;v\prime_{ZS}=\frac{v-{µ}_{D}}{\sigma_{D}}.$$

#### 3.3.2. Feature Dimension Reduction

**Figure 5:**
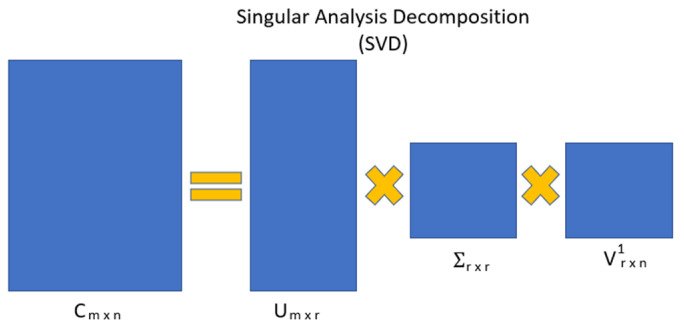
Singular value decomposition process for feature dimension reduction.

Feature dimension reduction refers to reducing the number of input variables for a predictive model. Note that simpler predictive models with fewer input variables may perform better when generating predictions based on new data [41]. For example, a matrix’s Singular Value Decomposition (SVD) is a factorization of linear algebra into three different matrices and transforms a dataset from its original dimension form into a new compressed dimension [42], as shown in Figure 5 and Equation (6).

The use of feature dimension reduction has been tested to discover how it affects categorization. The training time of the classifiers will be as short as possible since the number of observations is decreased after the dataset has been dimensionally reduced. Other than that, it communicates significant geometrical and theoretical insights regarding linear transformations, and it also has a few significant uses in data science.

(6) $$A=UWV^{T},$$

where

U: mxn matrix of the orthonormal eigenvectors of AA^^{T}^.

V^T^: transposition of an nxn matrix containing the orthonormal eigenvectors of A^^{T}^A.

W: an nxn diagonal matrix of the singular values, which are the square roots of the eigenvalues of A^^{T}^A.

#### 3.3.3. Feature Selection (Based on Time/Frequency Domain))

In this feature selection (Based on time/frequency domain) stage, the task is to select the best feature normalization method under the time or frequency domain. This method reduces input variables for the model and uses only relevant data [43]. The data have been obtained in terms of BN, DS, LS, MM, and ZS values. Apart from that, the features have been tested for their reliability by examining the classification accuracy with seven different classifiers, including Probabilistic Neural Network (PNN), Support Vector Machine (SVM), Naïve Bayes (NB), Decision Tree (DT), k-Nearest Neighbor (kNN), Discriminant Analysis (DA), and Ensemble (E). Subsequently, the selected feature domain continues to perform feature extraction.

#### 3.3.4. Feature Extraction

By generating new features from the current ones, feature extraction attempts to decrease the number of features in a dataset (and then discard the original features). The majority of the information in the original collection of features should then be summarized by this new, smaller set of features. Hence, combining the original set of features in this manner can produce a condensed version of the original features [44].

In this paper, only one domain feature is analyzed for the extraction stage. This helps to reduce the computation complexity and classification time. The features used in this study are Mean (M), Skewness (S), Standard Deviation (SD), Variance (V), Maximum FFT (Max FFT), and Minimum FFT (Min FFT), as shown in Equations (7)–(12).

M is the sum of values to the total number of values, as shown in Equation (7), where $v_{1}$ is the first value of data and *N* is the data sample size.

(7) $$\mathrm{Mean},\;\mu_{N}=\frac{v_{1}+v_{2}+v_{3}+\cdots +v_{N}}{N}.$$

S measures the asymmetry of a distribution, in which the distribution is symmetrical if it looks the same for both sides. Note that S is measured using Equation (8), where v is the data value, *N* is the data sample size, and $\mu_{N}$ is the mean.

(8) $$\mathrm{Skewness},\;\gamma_{N}=\frac{\sum \frac{\left(v-\mu_{N}\right)3}{N}}{{\left(\sum \frac{{\left(v-\mu_{N}\right)}^{2}}{N}\right)}^{\frac{3}{2}}}.$$

SD is used to measure the amount of variation of a set of values in data, as shown in Equation (9), where v*i* is the data value, *N* is the data sample size, and $\mu_{N}$ is the mean.

(9) $$\mathrm{Standard}\;\mathrm{deviation},\;\sigma_{N}=\sqrt{\sum \frac{{\left(v-\mu_{N}\right)}^{2}}{N}}.$$

V measures how far the value is from M. It is measured using Equation (10), where v is the data value, *N* is the data sample size, and $\mu_{N}$ is the mean.

(10) $$\mathrm{Variance},\;\sigma^{2}=\sum \frac{{\left(v-\mu_{N}\right)}^{2}}{N}.$$

Max FFT is the most significant value in a data set after transforming time domain data into frequency domain data using FFT. It is usually calculated using the max function in Matlab.

(11) $$\mathrm{Max}\;\mathrm{FFT}=v_{i}:v_{i}\geq v_{j},i\neq jAi,j\epsilon N.$$

Min FFT is the smallest value in a frequency domain data set and is calculated using the min function in Matlab.

(12) $$\mathrm{Min}\;\mathrm{FFT}=v_{i}:v_{i}\leq v_{j},i\neq jAi,j\epsilon N.$$

#### 3.3.5. Feature Selection (Optimization)

Feature selection (Optimization) reduces the number of input variables when developing a predictive model. In certain situations, reducing the number of input variables may increase the model’s efficiency while reducing the cost of modeling computations. Other than that, the relationship between each input variable and the target variable is evaluated using statistical feature selection techniques, and the input variables with the most robust relationships to the target variable are selected. Even though the choice of statistical measures is dependent on the data types of both the input and output variables, these techniques can be quick and effective [45,46,47].

This feature selection method is divided into two techniques, using the statistical method Analysis of Variance (ANOVA) and Binary Particle Swarm Optimization (BPSO) to select the best features. The ANOVA test determines the variance between the groups and the variance within the groups. Then, the data matrix must pass the selection criteria of a *p*-value less than 0.05, and the highest *p*-value is selected. 

After features undergo the ANOVA statistical test, a modified version of BPSO is used to analyze the best feature among the datasets. Note that BPSO sets the feature positions of a particle based on the discrete values of binary ‘0’ and ‘1’ values instead of continuous values [48,49]. A Sigmoid function is used to map the continuous-valued velocity given by Equation (13) to the range [0, 1], as shown in Equation (14) [45].

(13) $$sig\left(v_{id}\right)=\frac{1}{1+\exp\left(-v_{id}\right)}.$$

A particle’s feature states (i.e., positions) are changed based on the following equations. For example, the state of the *d*’th feature in particle *i* at time *t* is determined by [44]:

(14) $$x_{id}\left(t\right)=\left\{\begin{array}{c} 0,\;\;if\;\rho_{id}\;\geq \;sig\;\left(v_{id}\right)\\ 1,\;\;otherwise \end{array}\right.,$$

where $\rho_{id}$ is a random number with uniform distribution.

**Figure 6:**
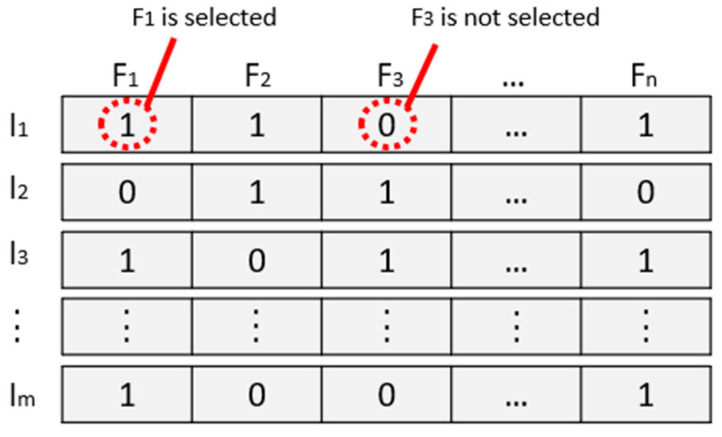
The BPSO process is represented by a particle.

The selected features from the previous step are utilized in the next stage. In the BPSO approach, each particle represents a string of binary bits 0–1 that specifies the features selected for inclusion in the subset, where “1” represents a feature that is selected, and “0” represents that it is not selected. Figure 6 shows an example of a solution represented by a particle, and the algorithm is outlined in Pseudocode BPSO in Feature Selection. Algorithm 1 express the Pseudocode BPSO in feature selection.
**Algorithm 1:** Pseudocode BPSO in Feature Selection.**Input: *n*—number of particles (swarm size);****   T—number of iteration;****   nVar—number of variables;****   Objective function;****Output:  Relevant features****1.   Start**2.   Initialize parameters of BPSO3.   Initialize the swarm**4.   Repeat**5.   **For** each particle **Do**6.   Evaluate particle’s fitness; (xbest)7.   Update particle’s neighborhood best position; (gbest)**8.   End**9.   **For** each particle **Do**10.    Update the particle’s velocity;11.    Update the particle’s position;**12.    End**13.    **Until** the stopping condition is true;14.    **End**

#### 3.3.6. Feature Fusion

**Figure 7:**
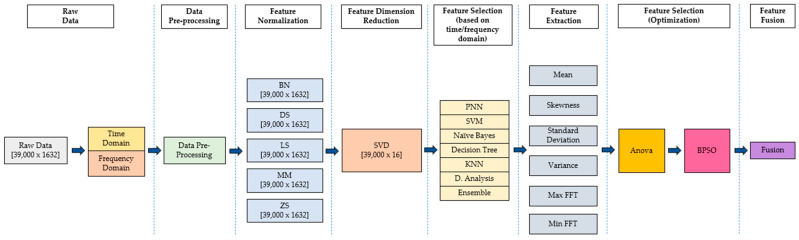
MSFS–BPSO framework.

Feature fusion is the hybridization of statistically selected features. In this stage, the selected features are fused to develop the proposed hybrid feature. Each dataset is reduced to a single column using the SVD method. At the end of this statistical feature MSFS–BPSO, a new hybrid feature is proposed. The novel framework of the proposed final design of the statistical feature generator is shown in Figure 7.

## 4. Results and Discussion

This section shows the results of the Multi-Stage Feature Selection with Binary Particle Swarm Optimization (MSFS–BPSO) process with the validation part. In feature dimension reduction, eight different dimensions have been tested, as shown in Table 3. In this test data sample, 39,000 rows of 1632 columns were compressed to 39,000 rows of 16 columns, giving the highest accuracy and minimizing the time below 12 s. Therefore, all data samples that continue for the next stage were be compressed to 39,000 rows and 16 columns. Reducing the dimensions of the original data indirectly increases the classification accuracy and reduces the processing time of the classifier.

In the feature selection (based on time/frequency domain) stage, the normalized–reduction datasets were tested using seven different classifiers to select a better result for the data sample, either in the time domain or frequency domain. Among the seven different classifiers used, the Probabilistic Neural Network (PNN) shows a stable result. The PNN classifiers can be tuned using the tunable parameter to optimize the classifier to better accuracy, shown in Table 4. In addition, the spread factor can be varied to control the degree of nonlinearity of the decision boundaries. Hence, it is the critical factor influencing the classifier’s classification performance. The spread factor for PNN has been varied in these experiments to obtain the best classification performance, which recorded existence (0.9), size (0.5), and location (0.01).

**Figure 8:**
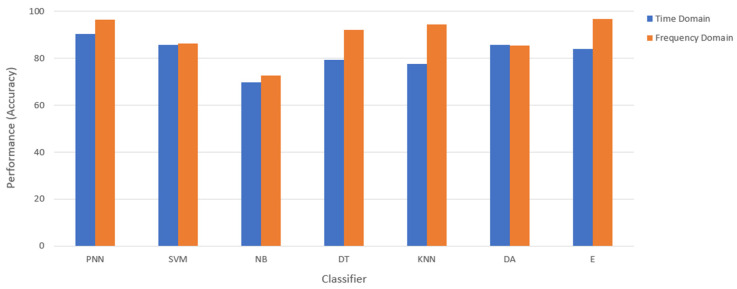
Comparison results between the time and frequency domain after feature normalization and feature dimension reduction.

The classification accuracy of each domain is clearly shown in Figure 8. The classification result has been obtained by averaging the classification accuracy for 50 repetitions. Most of the classifier shows that the frequency domain dominates compared to the time domain. Therefore, the data sample in the frequency domain was be selected for the next stage.

On the other hand, the feature extraction approach was used on the five normalized datasets stated in the previous section to carry out the feature selection procedure in the following stage. Six features made up of statistical combinations in the frequency domain were retrieved from each normalized dataset. The attributes are the following: M, SD, S,V, Max FFT, Min FFT. There are a total of 30 combinations of features from feature extraction as shown in Equations (15)–(19).

(15) $$\left[BN\right]=\left[M_{BN},S_{BN},SD_{BN},V_{BN},Max_{BN},Min_{BN}\right],$$

(16) $$\left[DS\right]=\left[M_{DS},S_{DS},SD_{DS},V_{DS},Max_{DS},Min_{DS}\right],$$

(17) $$\left[LS\right]=\left[M_{LS},S_{LS},SD_{LS},V_{LS},Max_{LS},Min_{LS}\right],$$

(18) $$\left[MM\right]=\left[M_{MM},S_{MM},SD_{MM},V_{MM},Max_{MM},Min_{MM}\right],$$

(19) $$\left[ZS\right]=\left[M_{ZS},S_{ZS},SD_{ZS},V_{ZS},Max_{ZS},Min_{ZS}\right]$$

**Figure 9:**
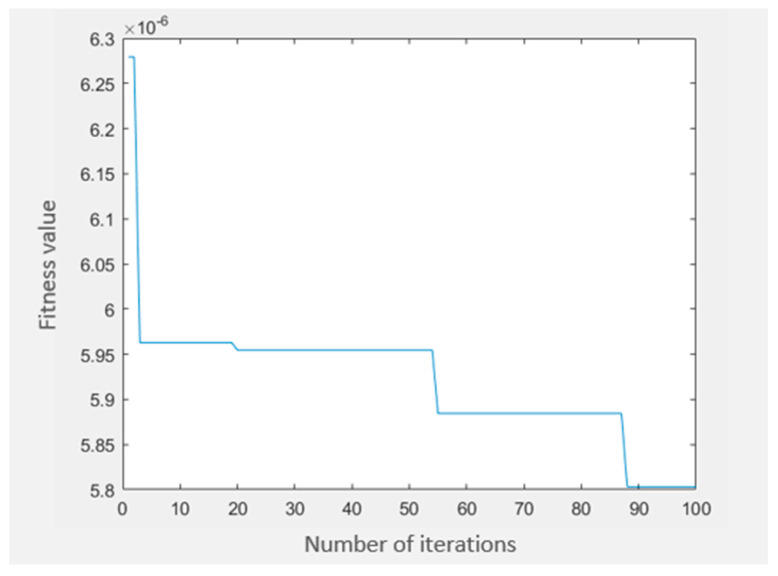
Convergence characteristic for BPSO in the global model.

From the extraction process, a total of 30 selected features were extracted. Note that the Analysis of Variance (ANOVA) process for *p*-value and f-value was calculated for each feature. All 30 features are less than 0.05 for *p*-values. Therefore, all 30 features were selected and rearranged accordingly from the highest f-value to the lowest, as shown in Table 5. Subsequently, Binary Particle Swarm Optimization (BPSO) suggested all possible hybrid feature datasets. For accuracy, it was tested using three different classifiers: PNN, Support Vector Machine (SVM), and k-Nearest Neighbor (kNN). The highest accuracy for the hybrid dataset is 14-HybridFeature (existence), 12-HybridFeature (size), and 16-HybridFeature (location). Figure 9 represents the convergence characteristic for BPSO in finding the optimum global model. The hyperparameter used in this BPSO is the following: swarmSize = 30, maxiter = 100, wMAX = 0.9 and wMIN = 0.2.

When a breast is screened for tumors at an early stage, there is a high risk of misclassification from a medical standpoint. Examining a few statistical measures obtained by calculating the classifier’s sensitivity, specificity, and accuracy scores allow for a classifier’s performance evaluation. Note that misclassification is when a tumor is present but not detected by the system or where no tumor is present, but the classifier detects a tumor. Such a possibility would negatively impact the system’s overall efficiency and must therefore be eliminated or minimized.

The sensitivity is calculated by dividing the number of correct selections by the total number of deserved selections, as shown in Equation (21). The particularity in Equation (22) represents the ratio of correctly rejected decisions to the total number of decisions that deserve rejection. Other than that, accuracy is the ratio of correct decisions to the total number of decisions made. TP represents true positive (indicates correct classification), FP represents false positive (indicates incorrect classification), *TN* represents true negative (indicates the incorrect classification of non-existence), and *FN* represents false negative (indicates the incorrect classification of non-existence). Equations (23)–(26) respectively state precision, recall, F1-measure and G-mean [50,51].

(20) $$Accuracy=\frac{TP+TN}{TP+TN+FP+FN}\times 100\%,$$

(21) $$\mathrm{Sensitivity}=\frac{TP}{TP+FN}\times 100\%,$$

(22) $$\mathrm{Specificity}=\frac{TN}{TN+FP}\times 100\%.$$

(23) $$Precision=\frac{TP}{TP+FP}.$$

(24) $$Recall=\frac{TP}{TP+FN}.$$

(25) $$F1=\frac{2}{\left(Precision^{-1}+Recall^{-1}\right)}.$$

(26) $$G-mean=\sqrt{\left(Precision\times Recall\right)}.$$

As seen in Table 6, the sensitivity, specificity, accuracy, precision, recall, F1-measure and G-mean of each classifier have been tabulated. The table shows that the dimensional reduction and fusion of the features to form hybrid features have deliberately increased the classification accuracy of the classifiers. The success rate of MSFS–BPSO-based hybrid features in the PNN classifier surpasses the performance of other common classifiers.

Table 7 compares the accuracy of the proposed system of MSFS–BPSO with another existing method. Most researchers used a small data set compared to this project, which is 80 times larger than an existing project. This is important for building analytic models with more extensive datasets using machine learning. From the result, the proposed MSFS–BPSO method is better than the other existing method, in which the proposed MSFS–BPSO method achieves 96.3%.

## 5. Conclusions

This study proposes a novel breast cancer classification framework that utilizes Multi-Stage Feature Selection with Binary Particle Swarm Optimization (MSFS–BPSO) using Ultra-Wideband (UWB).

The proposed framework has six stages. The first stage consists of feature normalization to change the feature to the same scale. The second stages consist of feature dimension reduction, which transforms the data from high dimensional space into low dimensional space without losing important properties of the original data. Next, feature selection (based on time/frequency domain) is used to choose the best group result between the time and frequency domains. Note that only one type of domain is selected to continue the process. Subsequently, feature extraction is performed to reduce the number of features in a dataset by creating new features from the existing one. After that, an optimized feature set is selected from the pool of new features using the Analysis of Variance (ANOVA)–BPSO technique. Finally, feature fusion combines different features from different layers and get them ready for analysis.

This study considered complete parameters in early breast cancer detection, including cancer existence, size detection, and location detection. The tumor models within the heterogenous breast phantom were classified, and their classification performance is as high as 96.3%, even though large data samples were fed into this model.

Other than that, the current research can be further improved with tests using various breast phantom structures, including various sizes and shapes (mimic of the actual breast), a more robust classification model, and comparing breast cancer detection using multiple UWB antennae.

## Figures and Tables

**Table 1:** Summary of the previous study on breast cancer detection using UWB.

Reference	Dataset	Classification Technique	Limitation
Lu et al., 2022 [26]	Breast phantom (6400 data sample)	A Convolutional Neural Network Long Short-Term (CNN-LSTM) network	Detection and quadrant localization only
Liu et al., 2021 [27]	Breast phantom (11,232 data sample)	SVM	Detection and localization
Vijayasarveswari et. al., 2020 [25]	Breast phantom (6750 data sample)	MSFS	Focus on size detection only
Bari et. al., 2020 [24]	Breast phantom (448 data sample)	FFBPNN	Limited data sample
Nouralhuda et. al., 2016 [22]	Breast phantom (118 data sample)	FFBPNN	Single feature extraction
Reeza et. al., 2015 [21]	Breast phantom (1632 data points)	Feature extraction and ANN	Only detect for existence and size Single feature technique
Alsheri et al., 2011 [28]	Breast phantom (6400 data points)	Feature extraction and ANN	Single feature technique

**Table 2:** Dielectric properties of breast phantom and tumor [25,28].

Breast Phantom	Material	Permittivity	Conductivity
Fatty tissues	Pure petroleum jelly	2.36	0.012
Glandular	Soy oil	2.7	0.061
Tumor	Mixture of water and wheat flour	6.98	0.785
Skin	Glass	3.5–10	Negligible

**Table 3:** Feature dimension reduction using SVD.

No.	Data Sample	Accuracy (%)	Time
1	1632 → 8	72.13	<10 s
**2**	**1632 → 16**	**86.35**	**<12 s**
3	1632 → 24	85.23	<12 s
4	1632 → 32	84.11	<14 s
5	1632 → 48	83.59	<15 s
6	1632 → 51	83.94	<15 s
7	1632 → 68	85.59	<16 s
8	1632 → 96	85.11	<18 s
9	(Raw → Data BN → SVM)	64.62	9 min 20 s
10	(Raw Data → SVM)	35.45	28 min 30 s

**Table 4:** PNN architecture is used in this work.

Parameters	Value for Existence	Value for Size	Value for Location
No. of input neurons	-	-	-
No. of output neurons	-	-	-
Spread factor	0.9	0.5	0.001
Testing tolerance	0.001	0.001	0.001
No. of training samples	13,650	11,700	11,700
No. of the validation sample	3900	1950	1950
No. of testing samples	6825	5850	5850
Total number of samples	39,000	19,500	19,500

**Table 5:** F-value for all features.

No.	Features	F-Value
1	Min_LS_	33,554.17903
2	Min_DS_	33,554.17697
3	Min_BN_	31,176.84962
4	Max_MM_	31,162.38708
5	M_ZS_	18,605.08924
6	Max_ZS_	3889.195375
7	SD_ZS_	3761.295512
8	Min_ZS_	3301.983306
9	S_ZS_	3180.094827
10	S_MM_	2585.315293
11	S_BN_	2445.623486
12	S_DS_	2168.639286
13	S_LS_	2168.639286
14	M_MM_	254.8622211
15	M_DS_	218.7147998
16	M_LS_	218.7147998
17	V_ZS_	209.6064994
18	M_BN_	207.9607835
19	SD_MM_	33.87221416
20	V_MM_	26.23552913
21	SD_BN_	24.28454284
22	SD_DS_	23.59648288
23	SD_LS_	23.59648288
24	Min_MM_	17.13184682
25	Max_BN_	12.48034536
26	V_BN_	12.10396575
27	V_LS_	11.44263249
28	V_DS_	11.44263248
29	Max_LS_	8.799857161
30	Max_DS_	8.799857155

**Table 6:** Average classification results in comparison between different classifiers for proposed hybrid features.

Parameter	Performance Evaluation	PNN	SVM	KNN
Existence	Accuracy (%)	98.25	97.48	97.50
	Sensitivity (%)	98.85	97.86	97.75
	Specificity (%)	96.23	95.48	95.22
	Precision	0.985	0.995	0.976
	Recall	0.99	0.98	0.985
	F1-measure	0.987	0.988	0.98
	G-mean	0.987	0.988	0.98
Size	Accuracy (%)	96.85	94.61	95.21
	Sensitivity (%)	95.35	94.48	96.62
	Specificity (%)	93.56	93.42	92.35
	Precision	0.975	0.966	0.984
	Recall	0.970	0.971	0.968
	F1-measure	0.973	0.969	0.976
	G-mean	0.973	0.969	0.96
Location	Accuracy (%)	93.81	92.23	91.49
	Sensitivity (%)	93.85	92.86	92.75
	Specificity (%)	92.87	91.71	90.82
	Precision	0.971	0.952	0.938
	Recall	0.926	0.966	0.961
	F1-measure	0.948	0.959	0.949
	G-mean	0.948	0.959	0.949

**Table 7:** Comparison results with the previous researcher.

Researcher	Data Sample	Method	Parameter	Test and Trains Sets	Accuracy
Vijayasarveswari [25]	6750	Multi-stage Feature Selection with Naïve Bayes classifier	Size	K-Fold Cross Validation	91.98%
Conceicao [23]	3744	PCA (feature extraction) with kNN classifier	Size and shape	K-Fold Cross Validation	96.2%
Bifta [24]	448	FFBPNN with feedforward net function	Existence, size, and location	70% Training 15% Validation 15% Testing	92.43%
Proposed work	39,000	Multi-stage Feature Selection (MSFS) with BPSO	Existence, size, and location	K-Fold Cross Validation	96.3%

## Data Availability

The data used in this manuscript is collected by author in Universiti Malaysia Pahang. 39,000 data sample used in this project.
